# Efficacy and safety of B/F/TAF in treatment-naïve and virologically suppressed people with HIV ≥ 50 years of age: integrated analysis from six phase 3 clinical trials

**DOI:** 10.1186/s12879-025-11476-3

**Published:** 2025-08-22

**Authors:** Cissy M. Kityo, Samir K. Gupta, Princy N. Kumar, Amy R. Weinberg, Bhumi Gandhi-Patel, Hui Liu, Jason T. Hindman, Jürgen K. Rockstroh

**Affiliations:** 1 Joint Clinical Research Centre, Kampala, Uganda; 2 Indiana University School of Medicine, Indianapolis, IN USA; 3 Georgetown University Medical Center, Washington, DC USA; 4 Gilead Sciences, Inc, Foster City, CA USA; 5 University Hospital Bonn, Bonn, Germany

**Keywords:** HIV, Older adults, B/F/TAF, Antiretroviral therapy, Comorbidities

## Abstract

**Introduction:**

Older adults with HIV, particularly those ≥ 50 years of age, face unique health challenges due to a higher prevalence of comorbidities and polypharmacy, which can impact medication adherence and increase the risk of adverse events. We assessed the efficacy and safety of bictegravir/emtricitabine/tenofovir alafenamide (B/F/TAF) in people with HIV (PWH) ≥ 50 years of age across treatment-naïve and virologically suppressed cohorts over a long-term follow-up.

**Methods:**

This post hoc analysis included participants ≥ 50 and < 50 years of age from six phase 3 trials of B/F/TAF, comprising 2 treatment-naïve studies and 4 virologically suppressed studies. Outcomes were assessed through Week 240 for the treatment-naïve cohort and Week 48 for the virologically suppressed cohort. Key measures included virologic outcomes (HIV-1 RNA < 50 or ≥ 50 copies/mL), CD4 T-cell changes, adherence, metabolic and renal parameters, treatment-emergent adverse events, and treatment-emergent diabetes and hypertension.

**Results:**

The treatment-naïve cohort included 96 participants ≥ 50 years of age and 538 participants < 50 years of age, while the virologically suppressed cohort included 450 participants ≥ 50 years of age and 640 participants < 50 years of age. By Week 240, virologic suppression was achieved in 98.5% of treatment-naïve participants ≥ 50 years of age and in 98.6% of those < 50 years of age, as determined using missing = excluded analysis. By Week 48, virologic failure was 0.9% versus 1.4% in participants ≥ 50 years of age versus < 50 years of age, respectively, and virologic suppression was maintained in 93.6% of virologically suppressed participants in both the ≥ 50 and < 50 years of age groups, as assessed using the US Food and Drug Administration snapshot algorithm. Across age groups, the treatment-naïve and virologically suppressed cohorts demonstrated comparable outcomes beyond viral load through Weeks 240 and 48, respectively, including CD4 T-cell changes, adherence rates of ≥ 95%, body weight, lipid profiles, renal function, bone health, treatment-emergent adverse events, and the incidence of treatment-emergent diabetes and hypertension.

**Conclusions:**

These results highlight the durability, long-term efficacy, safety, and overall benefits of B/F/TAF in PWH ≥ 50 years of age.

**Supplementary Information:**

The online version contains supplementary material available at 10.1186/s12879-025-11476-3.

## Introduction

Antiretroviral therapy (ART) has greatly improved the life expectancy of people with HIV; PWH), changing the demographic characteristics of the epidemic [1, 2]. Presently, an estimated 24% of the global HIV-positive population is ≥ 50 years of age [3]. As the global population of PWH ≥ 50 years of age continues to increase, so does the need for ART regimens that address the unique health challenges of this aging cohort [4–8]. Older PWH frequently experience higher rates of age-related comorbidities, such as cardiovascular disease, diabetes, and hypertension, which is often exacerbated by long-term ART exposure; these comorbidities, combined with prolonged ART use, increase the risk of adverse drug events and present specific challenges in managing bone health, renal function, metabolic parameters, cardiovascular health, and other conditions [9–15].

Bictegravir/emtricitabine/tenofovir alafenamide (B/F/TAF) is a single-tablet regimen that has shown efficacy and safety in treatment-naïve participants and those who are virologically suppressed [16–18]. Its formulation, which includes tenofovir alafenamide (TAF), offers improved renal and bone safety profiles compared with tenofovir disoproxil fumarate (TDF)–based treatments, which is particularly relevant for aging adults with comorbidities [19–21]. However, data on the long-term use of B/F/TAF in older adults remain limited, especially regarding its efficacy and safety in managing the complex health profiles of PWH ≥ 50 years of age.

To address this gap, our study pools data from six phase 3 clinical trials to evaluate the long-term efficacy and safety of B/F/TAF in participants ≥ 50 years of age, encompassing both treatment-naïve and virologically suppressed populations. By focusing on this demographic, our study aims to inform best practices for managing HIV in aging populations and to reduce disparities in health outcomes for older PWH.

## Methods

### Overall study design and participants (supplemental fig. 1)

This post hoc analysis utilized data from 2 cohorts across six phase 3 clinical trials to evaluate the long-term efficacy and safety of B/F/TAF in PWH ≥ 50 years of age. The study included participants who were both treatment-naïve and virologically suppressed. For the treatment-naïve cohort, participants were drawn from B/F/TAF group (randomized to B/F/TAF) of Studies 1489 (ClinicalTrials.gov Identifier: NCT02607930; Clinical trial number: not applicable) and 1490 (NCT02607956). In these trials, eligible participants had plasma HIV-1 RNA levels ≥ 500 copies/mL at screening and were ART-naïve. Study 1489 excluded individuals with chronic hepatitis B virus (HBV) infection or HLA-B*5701 positivity and required an estimated glomerular filtration rate (eGFR) ≥ 50 mL/min, while Study 1490 allowed participants with chronic HBV or hepatitis or C virus infection and required an eGFR ≥ 30 mL/min. The methods for both studies have been previously published [16, 19].

For the virologically suppressed cohort, participants were selected from the B/F/TAF group (randomized to B/F/TAF) of Studies 1844 (NCT02603120), 1878 (NCT02603107), 1961 (NCT02652624), and 4030 (NCT03110380). Specifically, Study 1844 did not require stable suppression (< 50 copies/mL) for ≥ 6 months before switching; whereas, Study 4030 included participants with documented suppression of HIV-1 RNA < 50 copies/mL for a minimum period of ≥ 3 or ≥ 6 months depending on the preceding treatment regimen (dolutegravir + emtricitabine/TAF or dolutegravir + emtricitabine/TDF) and the assay sensitivity (with a threshold of ≥ 50 copies/mL, as applicable). Throughout the studies, virologically suppressed participants were required to maintain viral suppression and were excluded if they modified lipid-lowering medications or other ART regimens that could influence study outcomes.

All the studies were conducted in accordance with the Declaration of Helsinki and approved by the US Food and Drug Administration (FDA). Institutional review boards at each site granted ethical approval, and all participants provided written informed consent.

### Study outcomes and assessments

This analysis reports efficacy and safety outcomes among participants ≥ 50 years of age in 2 cohorts across 6 studies that assessed B/F/TAF treatment. The treatment-naïve cohort included participants who were randomized to B/F/TAF and received B/F/TAF up to Week 240 (end of study). For the virologically suppressed cohort, the studies included participants who were randomized to B/F/TAF and who received B/F/TAF up to the Week 48 analysis data cutoff of the randomized phase.

In all 6 studies, plasma HIV-1 RNA levels were measured using the Roche TaqMan™ 2.0 assay (Roche Diagnostics, Rotkreuz, Switzerland), and CD4 T-cell counts were assessed at baseline (Day 1) followed by Weeks 4, 8, and 12, and subsequently every 12 weeks throughout the randomized phase. Additionally, lipid profiles (total cholesterol [TC], high-density lipoprotein [HDL] cholesterol, direct low-density lipoprotein [LDL] cholesterol, and triglycerides) were measured after an 8-hour fast at baseline, and assessments were conducted at Weeks 12, 24, 48, 72, 96, 120, and 144 for the treatment-naïve cohort, with continued assessments every 12 weeks. For the virologically suppressed cohort, assessments were conducted at Weeks 12, 24, and 48.

The efficacy outcomes included the proportion of participants with plasma HIV-1 RNA < 50 copies/mL at Week 240 for the treatment-naïve cohort, which was analyzed using missing = excluded (M = E) and missing = failure (M = F) methods (with imputation of missing data as exclusion and failure, respectively), and the proportion of participants with plasma HIV-1 RNA < 50 or ≥ 50 copies/mL at Week 48 for the virologically suppressed cohort, which was analyzed primarily using the FDA snapshot method (with the proportion of participants with plasma HIV-1 RNA < 50 copies/mL at Week 48 by M = E and M = F methods as other efficacy outcomes). Change in CD4 T-cell count from baseline to Week 240 (for the treatment-naïve cohort) and to Week 48 (for the virologically suppressed cohort) was also another efficacy outcome.

Other outcomes focused on safety, examining changes in renal function (eGFR) and bone mineral density (BMD) as indicators of long-term treatment impact. BMD was measured using dual x-ray absorptiometry scans, which were available at Week 240 for the treatment-naïve cohort in Study 1489 and at Week 48 for the virologically suppressed cohorts. Metabolic parameters, including changes in body weight and fasting lipid levels, were assessed to identify any treatment-emergent metabolic effects. Treatment-emergent adverse events (TEAEs) and new-onset diabetes mellitus, hypertension, and any treatment-emergent viral resistance were monitored. Adherence to the B/F/TAF regimen was assessed through pill counts, with adherence rates ≥ 95% classified as high and < 85% classified as low.

The analysis also accounted for metabolic comorbidities and renal outcomes, which were reported using standardized *Medical Dictionary for Regulatory Activities* query (SMQ; version 24.0 for the treatment-naïve cohort and version 26.1 for the virologically suppressed cohort) search lists through Week 240 for the treatment-naïve cohort or through Week 48 for the virologically suppressed cohort. New diagnoses of diabetes mellitus and hypertension were identified via the hyperglycemia/new-onset diabetes mellitus SMQ (narrow scope) and hypertension SMQ, respectively. Participants with a history of diabetes or hypertension at baseline were excluded from the respective treatment-emergent diabetes or hypertension assessments.

### Statistical analysis

Baseline demographic and clinical characteristics were summarized using descriptive statistics, including means and standard deviations (SDs) for continuous variables and proportions for categorical variables. The proportion of participants with plasma HIV-1 RNA < 50 copies/mL at Week 240 for the treatment-naïve cohort using M = E and M = F methods was summarized. The difference in the percentage of participants with HIV-1 RNA < 50 copies/mL at Week 240 between age groups and its 95% confidence interval (CI) were calculated based on Mantel-Haenszel proportions and adjusted by baseline HIV-1 RNA stratum (≤ 100000 vs. > 100000 copies/mL) and region stratum (US vs. Ex-US). The difference in the percentage of participants with HIV-1 RNA ≥ 50 or < 50 copies/mL at Week 48 by snapshot algorithm between age groups and its 95% CI was calculated using an unconditional exact method with 2 inverted 1-sided tests. *P* values for comparing the 2 age groups were derived from the Fisher exact test. In general, no adjustment for multiplicity was made for outcomes evaluated in this post hoc analysis. Nominal *P* values (*P* values without multiplicity adjustment) were provided.

Change in CD4 T-cell count from baseline to Week 240 for the treatment-naïve cohort and to Week 48 for the virologically suppressed cohort was compared between age groups using an analysis of variance model, with age group as a fixed effect. Between treatment groups, the difference in adherence categories (≥ 95%, ≥ 85 to < 95%, and < 85%) was compared using the Cochran-Mantel-Haenszel statistics test.

TEAEs, fasting lipids, weight changes, and metabolic outcomes, including treatment-emergent diabetes mellitus and hypertension, were analyzed descriptively. In addition, for continuous metabolic and renal outcomes, such as fasting lipids (TC, LDL, HDL, triglycerides, and TC ratio) and weight changes from baseline, the 2-sided Wilcoxon rank sum test was used for age group comparison of continuous variables. Fisher’s exact test was applied for age group comparison of categorical outcomes, including treatment-emergent diabetes mellitus and hypertension. Safety data were summarized, covering all data from the first dose of study drug to the data cutoff date or up to 30 days after treatment discontinuation, if applicable. All statistical analysis were conducted using SAS software (version 9.4).

## Results

### Participant demographic and baseline characteristics

In this pooled analysis of 2 cohorts from 6 studies that assessed B/F/TAF in participants ≥ 50 years of age (compared with participants < 50 years of age), baseline characteristics were evaluated across both treatment-naïve and virologically suppressed cohorts (Table 1).

**Table 1:** Demographics and characteristics

		Treatment-naïve cohort		Virologically suppressed cohort	
Median (Q1, Q3) age, years		≥50 years	<50 years	≥50 years	<50 years
		*n* = 96	*n* = 538	*n* = 450	*n* = 640
		55 (52, 60)	30 (25, 37)	56 (52,60)	39 (33,45)
Male sex at birth, n (%)		81 (84.4)	484 (90.0)	342 (76.0)	393 (61.4)
Region, n (%)	US	56 (58.3)	365 (67.8)	327 (72.7)	294 (45.9)
	Ex-US	40 (41.7)	173 (32.2)	123 (27.3)	346 (54.1)
Race, n (%)	White	59 (61.5)	304 (56.5)	291 (64.7)	369 (57.7)
	Black	30 (31.3)	181 (33.7)	131 (29.1)	166 (25.9)
	Other^a^	4 (4.2)^b^	36 (6.7)^b^	17 (3.8)^b^	38 (5.9)^b^
Hispanic or Latino ethnicity, n (%)		11 (11.5)	144 (26.9)^c^	72 (16.0)^c^	131 (20.5)^d^
HIV-1 RNA log_10_ c/mL (Q1, Q3)		4.5 (4.0, 4.9)	4.4 (4.0, 4.9)	N/A	N/A
HIV-1 RNA < 50 c/mL, n (%)		N/A	N/A	441 (98.0)	632 (98.8)
HIV-1 RNA > 100,000 c/mL, n (%)		23 (24.0)	96 (17.8)	0	0
CD4 T-cell count, cells/µL, median (IQR)		436 (235–601)	442 (299–590)	640 (486–852)	691 (523–887)
Medical history, n (%)	Cardiovascular disease	6 (6.3)	8 (1.5)	50 (11.1)	15 (2.3)
	Diabetes mellitus	16 (16.7)	22 (4.1)	76 (16.9)	38 (5.9)
	Hyperlipidemia	39 (40.6)	48 (8.9)	220 (48.9)	121 (18.9)
	Hypertension	46 (47.9)	52 (9.7)	182 (40.4)	111 (17.3)

*c* copies, *HIV-1* Human immunodeficiency virus–1, *IQR* Interquartile range, *N/A* Not applicable

^a^Includes American Indian or Alaska Native, Asian, Native Hawaiian or Pacific Islander, and other

^b^Race data were not available for 1 participant

^c^Ethnicity data were not available for 1 participant

^d^Ethnicity data were not available for 2 participants

Among treatment-naïve participants, the median age was 55 years (quartile [Q]1, Q3: 52, 60) for those ≥ 50 years of age (*n* = 96) and 30 years (Q1, Q3: 25, 37) for those < 50 years of age (*n* = 538). This cohort included 5 participants ≥ 65 years of age, 91 participants 50 to < 65 years of age, and 538 participants < 50 years of age, for a total of 634 participants in the B/F/TAF analysis set. The majority of participants were male at birth, with 84.4% in the ≥ 50 years of age group and 90.0% in the < 50 years of age. Regionally, 58.3% of participants ≥ 50 years of age and 67.8% of those < 50 years of age were from the United States, with the remainder from other countries. Racially, 61.5% of participants ≥ 50 years of age and 56.5% of those < 50 years of age identified as White, while 31.3% and 33.7%, respectively, identified as Black. Hispanic or Latino ethnicity was reported by 11.5% of participants ≥ 50 years of age and 26.9% of those < 50 years of age. Median baseline HIV-1 RNA level was 4.48 log_10_ copies/mL (Q1, Q3: 4.00, 4.93) for participants ≥ 50 years of age and 4.41 log_10_ copies/mL (Q1, Q3: 4.00, 4.86) for those < 50 years of age. CD4 T-cell counts were similar, with a median of 436 cells/µL (Q1, Q3: 235, 601) in the ≥ 50 years of age group and 442 cells/µL (Q1, Q3: 299, 590) in the < 50 years of age group. Among participants ≥ 50 years of age, 16.7% had diabetes, 6.3% had cardiovascular disease, 40.6% had hyperlipidemia, and 47.9% had hypertension. Among participants < 50 years of age, 4.1% had diabetes, 1.5% had cardiovascular disease, 8.9% had hyperlipidemia, and 9.7% had hypertension (Table 1).

For the virologically suppressed cohort, the median age was 56 years (Q1, Q3: 52, 60) for participants ≥ 50 years of age (*n* = 450) and 39 years (Q1, Q3: 33, 45) for those < 50 years of age (*n* = 640). This cohort included 54 participants ≥ 65 years of age, 396 participants 50 to < 65 years of age, and 640 participants < 50 years of age, for a total of 1090 in the B/F/TAF safety analysis set. Most participants were male at birth, with 76.0% in the ≥ 50 years of age group and 61.4% in the < 50 years of age group. In terms of region, 72.7% of those ≥ 50 years of age and 45.9% of those < 50 years of age were from the United States. Among those ≥ 50 years of age, 64.7% of participants identified as White, 29.1% identified as Black, and 16.0% reported Hispanic or Latino ethnicity. In the < 50 years of age group, 57.7% of participants identified as White, 25.9% identified as Black, and 20.5% reported Hispanic or Latino ethnicity. Baseline virologic suppression was high, with 98.0% (441/450) of those ≥ 50 years of age and 98.8% (632/640) of those < 50 years of age having HIV-1 RNA < 50 copies/mL. Median CD4 T-cell counts were 640 cells/µL (Q1, Q3: 486, 852) for participants ≥ 50 years of age and 691 cells/µL (Q1, Q3: 523, 887) for those < 50 years of age. Medical history showed a notable prevalence of comorbidities among those ≥ 50 years of age: 11.1% of participants had cardiovascular disease, 16.9% had diabetes, 48.9% had hyperlipidemia, and 40.4% had hypertension. Among those < 50 years of age, 2.3% of participants had cardiovascular disease, 5.9% had diabetes, 18.9% had hyperlipidemia, and 17.3% had hypertension (Table 1).

### Virologic Outcomes

**Fig. 1:**
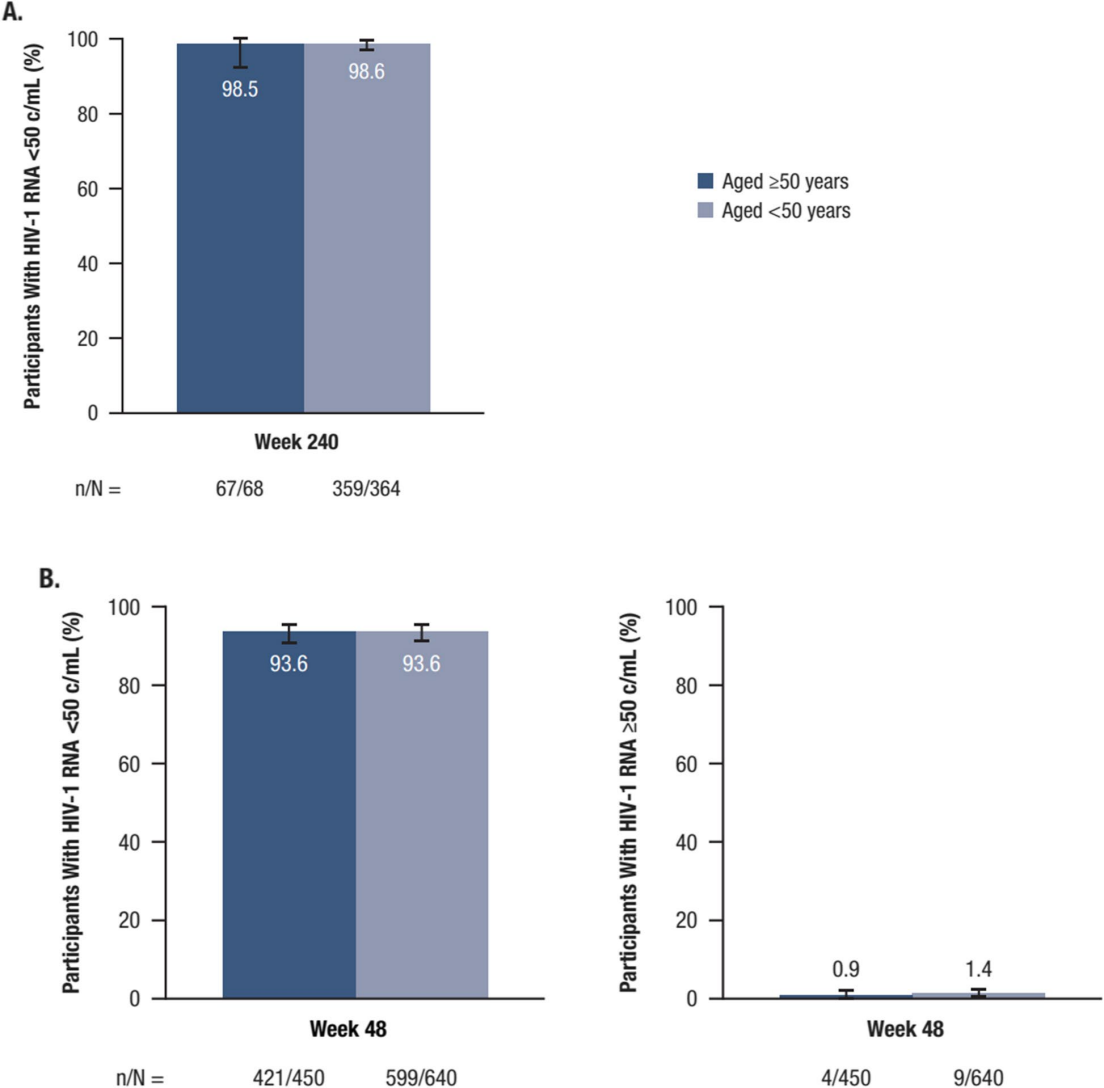
Virologic outcomes (M = E) in the treatment-naïve (**A**) and virologically suppressed (**B**) cohorts. B/F/TAF, bictegravir/emtricitabine/tenofovir alafenamide; c, copies; HIV-1, human immunodeficiency virus–1; M = E, missing = excluded. Results are shown using the M=E approach unless otherwise indicated. **A** Treatment-naïve cohort: the rate of virologic suppression (HIV-1 RNA<50 c/mL) with B/F/TAF was similar at Week 240 between age groups (M = E). **B** Virologically suppressed cohort: the rate of virologic failure (HIV-1 RNA ≥50 c/mL) was low and virologic suppression (HIV-1 RNA <50 c/mL) was high with B/F/TAF at Week 48 in both age groups (FDA Snapshot)

In the treatment-naïve cohort, virologic suppression (HIV-1 RNA < 50 copies/mL) at Week 240 was achieved by 98.5% of participants (67/68) ≥ 50 years of age (95% CI: 92.1%−100.0%) and by 98.6% of participants (359/364) < 50 years of age (95% CI: 96.8%−99.6%), as determined by the M = E analysis (*P* = 0.9139; Fig. 1A). In the M = F analysis, virologic suppression at Week 240 was maintained by 69.8% of participants (67/96) ≥ 50 years of age (95% CI: 59.6%−78.7%) and by 66.7% of participants (359/538) < 50 years of age (95% CI: 62.6%−70.7%), with no significant differences observed between the 2 age groups (*P* = 0.50).

At Week 48 in the virologically suppressed cohort, virologic suppression (HIV-1 RNA < 50 copies/mL), as assessed by the FDA snapshot method, was achieved by 93.6% of participants, with 93.6% of participants (421/450; 95% CI: 90.9%−95.6%) ≥ 50 years of age and 93.6% of participants (599/640; 95% CI: 91.4%−95.4%) < 50 years of age achieving suppression; there were no statistically significant differences between the 2 groups (*P* = 1.00; Fig. 1B). The proportion of participants with HIV-1 RNA ≥ 50 copies/mL was 0.9% (4/450; 95% CI: 0.2%−2.3%) for participants ≥ 50 years of age and 1.4% (9/640; 95% CI: 0.6%−2.7%) for those < 50 years of age, with no statistically significant differences between the 2 groups (*P* = 0.58; Fig. 1B). At Week 48, virologic suppression (HIV-1 RNA < 50 copies/mL) was achieved by 99.3% of participants (426/429; 95% CI: 98.0%−99.9%) ≥ 50 years of age and 98.7% of participants (602/610; 95% CI: 97.4%−99.4%) < 50 years of age in the M = E analysis. The difference between the 2 age groups was not statistically significant (*P* = 0.54). At Week 48, virologic suppression (HIV-1 RNA < 50 copies/mL) was achieved by 94.7% of participants (426/450; 95% CI: 92.2%−96.6%) ≥ 50 years of age and 94.1% of participants (602/640; 95% CI: 91.9%−95.8%) < 50 years of age in the M = F analysis. The difference between the 2 age groups was not statistically significant (*P* = 0.69). No treatment-emergent resistance to B/F/TAF was observed in either age group through Week 240 in the treatment-naïve cohort or through Week 48 in the virologically suppressed cohort.

### CD4 T-cell counts 

At Week 240 in the treatment-naïve cohort, CD4 T-cell counts continued to increase from baseline in both age groups (mean [SD] change from baseline: +291 [221.3] for participants ≥ 50 years of age and + 347 [238.2] for those < 50 years of age; least squares mean difference [LSMD]: − 58 [range: − 120, 4]; *P* = 0.07). At Week 48 in the virologically suppressed cohort, CD4 T-cell count also increased from baseline among participants ≥ 50 years of age and those < 50 years of age (mean [SD] change from baseline: +18 [162.5] and + 4 [174.9] cells/µL, respectively; LSMD: 15 [range: − 7, 36]; *P* = 0.18), indicating no statistically significant difference between age groups.

### Adherence

Adherence to B/F/TAF through Week 240 for the treatment-naïve cohort and through Week 48 for the virologically suppressed cohort is shown in Table 2. In the treatment-naïve cohort, the median adherence rate was high in both age groups, with participants ≥ 50 years of age showing a median (Q1, Q3) adherence of 98.2% (96.7, 99.4) and those < 50 years of age at 97.0% (93.2, 98.9). A greater proportion of participants ≥ 50 years of age achieved an adherence rate of ≥ 95% compared with those < 50 years of age (82.8% vs. 66.3%, respectively; *P* = 0.002; Table 2). In the virologically suppressed cohort, the median adherence rate was also high for both age groups at Week 48, with a median (Q1, Q3) adherence of 98.8% (96.8, 99.7) for participants ≥ 50 years of age and 98.8% (96.8, 99.7) for those < 50 years of age. The proportion of participants with adherence rates of ≥ 95%, ≥ 85 to < 95%, and < 85% was similar between age groups, indicating consistent adherence across age categories in the virologically suppressed cohort (Table 2).

**Table 2:** Adherence

	Treatment-naïve cohort		Virologically suppressed cohort	
	≥ 50 years of age	< 50 years of age	≥ 50 years of age	< 50 years of age
	*n* = 96	*n* = 538	*n* = 450	*n* = 640
Participants who returned ≥ 1 bottle, n (%)	93 (96.9)	531 (98.7)	448 (99.6)	639 (99.8)
Median (Q1, Q3) adherence, %	98.2 (96.7, 99.4)	97.0 (93.2, 98.9)	98.8 (96.8, 99.7)	98.8 (96.8, 99.7)
< 85%, n (%)	5 (5.4)	39 (7.3)	11 (2.5)	15 (2.3)
≥ 85% to < 95%, n (%)	11 (11.8)	140 (26.4)	62 (13.8)	79 (12.4)
≥ 95%, n (%)	77 (82.8)	352 (66.3)	375 (83.7)	545 (85.3)

Adherence was calculated based on pill count for the study drug (B/F/TAF). Denominator for the percentage of drug adherence was the number of participants who returned ≥ 1 bottle and had calculable drug adherence. For the treatment-naïve cohort, adherence was measured through Week 240 (end of study). For the virologically suppressed cohort, adherence was measured through Week 48

*B/F/TAF* Bictegravir/emtricitabine/tenofovir alafenamide, *Q* Quartile

### Outcomes in body weight, lipid profile, renal function, and bone health

Change from baseline in body weight through Week 240 is shown for participants ≥ 50 and < 50 years of age in the treatment-naïve cohort and through Week 48 for the virologically suppressed cohort. In the treatment-naïve cohort, there were no significant overall differences in weight change at Week 240 between participants ≥ 50 years of age and those < 50 years of age. The median (Q1, Q3) weight gain was 4.8 kg (0.7, 10.2) for participants ≥ 50 years of age and 6.4 kg (2.4, 12.0) for those < 50 years of age. At Week 48 in the virologically suppressed cohort, change in body weight was minimal, with a median (Q1, Q3) weight gain of 1.5 kg (–0.8, 3.8) for participants ≥ 50 years of age and 1.8 kg (–0.4, 4.0) for those < 50 years of age. No significant difference in weight change was observed between age groups in either cohort (Table 3 and Supplement Table 1).

**Table 3:** Bone, renal, and metabolic outcomes

		Treatment-naïve cohort					Virologically suppressed cohort					
		≥ 50 years of age	N	< 50 years of age	N	*P* value		≥ 50 years of age	N	< 50 years of age	N	*P* value
Median body weight, kg (Q1, Q3)	Baseline	79.3 (70.7, 89.9)	96	75.9 (67.3, 87.1)	538	0.0285	Baseline	81.1 (71.9, 92.5)	450	76.0 (66.0, 87.1)	640	< 0.0001
	Change at Week 240	4.8 (0.7, 10.2)	68	6.4 (2.4, 12.0)	363	0.087	Change at Week 48	1.5 (–0.8, 3.8)	429	1.8 (–0.4, 4.0)	609	0.2857
Median TC: HDL ratio (Q1, Q3)^a^	Baseline	4.1 (3.2, 5.0)	93	3.7 (3.0, 4.5)	526	0.0017	Baseline	3.7 (3.1, 4.5)	444	3.7 (3.1, 4.6)	630	0.9738
	Change at Week 240	–0.3 (–0.9, 0.4)	65	0.1 (–0.4, 0.6)	345	0.0044	Change at Week 48	–0.1 (–0.5, 0.4)	412	0.0 (–0.4, 0.3)	593	0.933
Median eGFR, mL/min (Q1, Q3)^b^	Baseline	99.2 (83.6, 114.0)	96	126.3 (108.5, 146.8)	538	< 0.0001	Baseline	88.6 (75.5, 105.4)	450	107.9 (93.0, 126.6)	640	< 0.0001
	Change at Week 240	–10.5 (–19.6, 2.4)	67	–7.7 (–19.4, 3.0)	363	0.3003	Change at Week 48	–0.9 (–8.1, 5.8)	422	–1.0 (–9.6, 8.4)	603	0.9223

Baseline value was defined as the last nonmissing value obtained on or prior to the first dose of B/F/TAF. *P* values were from the 2-sided Wilcoxon rank sum test

*B/F/TAF* Bictegravir/emtricitabine/tenofovir alafenamide, *eGFR* Estimated glomerular filtration rate, *HDL* High-density lipoprotein, *Q* Quartil, TC Total cholesterol

^a^Only laboratory measurements under fasting conditions are summarized. ^b^By Cockcroft-Gault equation

Change from baseline in fasting lipid parameters, specifically the TC: HDL ratio, was assessed across both age groups. At Week 240 in the treatment-naïve cohort, the median (Q1, Q3) change from baseline in TC: HDL ratio was − 0.3 (–0.9, 0.4) for participants ≥ 50 years of age and 0.1 (–0.4, 0.6) for those < 50 years of age. At Week 48 in the virologically suppressed cohort, change in the TC: HDL ratio was minimal, with a median (Q1, Q3) change of − 0.1 (–0.5, 0.4) for participants ≥ 50 years of age and 0.0 (–0.4, 0.3) for those < 50 years of age (Table 3). Additionally, the proportion of participants who initiated lipid-modifying agents during the study was higher among those ≥ 50 years of age compared with those < 50 years of age (5.3% vs. 0.8%, respectively; *P* < 0.0001).

Change from baseline in eGFR was similar across age groups in both the treatment-naïve and virologically suppressed cohorts. At Week 240 in the treatment-naïve cohort, the median (Q1, Q3) change in eGFR from baseline was − 10.5 mL/min (–19.6, 2.4) for participants ≥ 50 years of age and − 7.7 mL/min (–19.4, 3.0) for those < 50 years of age, with no statistically significant difference between age groups (*P* = 0.30). At Week 48 in the virologically suppressed cohort, the median (Q1, Q3) change in eGFR was − 0.9 mL/min (–8.1, 5.8) for participants ≥ 50 years of age and − 1.0 mL/min (–9.6, 8.4) for those < 50 years of age (*P* = 0.92), indicating minimal change and no significant difference across age groups (Table 3).

**Fig. 2:**
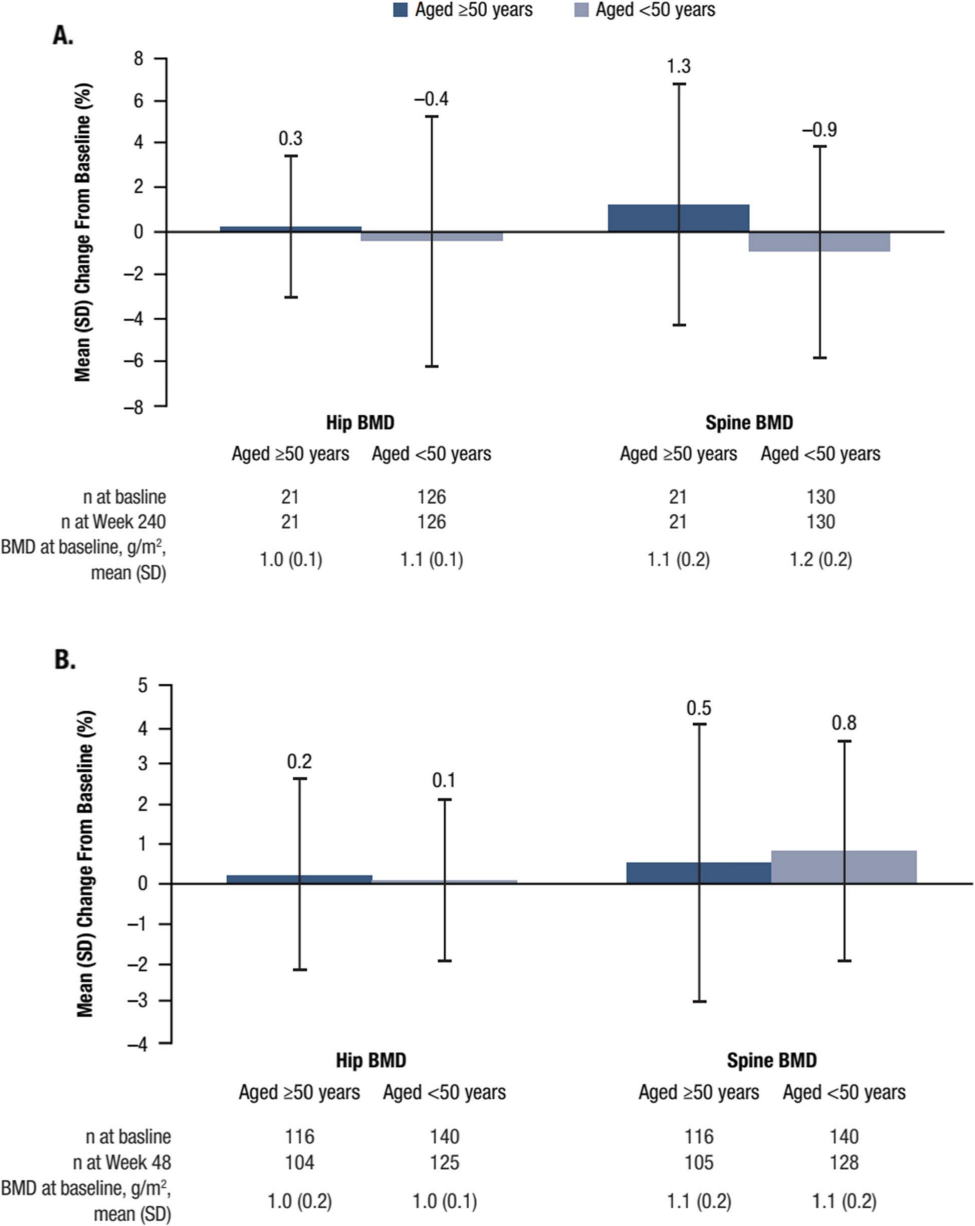
Percent change from baseline in BMD in the treatment-naïve (**A**) and virologically suppressed (**B**) cohorts. BMD, bone mineral density; SD, standard deviation. **A** Treatment-naïve cohort: mean percent change in hip and spine BMD from baseline to Week 240 in participants ≥50 years of age and <50 years of age. **B** Virologically suppressed cohort: mean percent change in hip and spine BMD from baseline to Week 48 in participants≥50 years of age and <50 years of age

Change from baseline in BMD was minimal and similar between age groups in both cohorts. At Week 240 in the treatment-naïve cohort, the mean (SD) percent change in hip BMD was 0.3% (3.26%) for participants ≥ 50 years of age and − 0.4% (5.80%) for those < 50 years of age, while mean (SD) percent change in spine BMD was 1.3% (5.64%) and − 0.9% (4.95%), respectively. At Week 48 in the virologically suppressed cohort, the mean (SD) percent change in hip BMD was 0.2% (2.43%) for participants ≥ 50 years of age and 0.1% (2.03%) for those < 50 years of age; mean percent change in spine BMD increased by 0.5% (3.52%) and 0.8% (2.78%), respectively. No significant difference was observed between age groups in either cohort (Fig. 2).

### Other safety outcomes

In the treatment-naïve cohort, TEAEs were reported by 93.8% of participants ≥ 50 years of age and by 95.5% of those < 50 years of age, with study drug–related TEAEs in 26.0% and 28.4% of participants, respectively. Grade 3 or 4 TEAEs affected 31.3% of participants ≥ 50 years of age and 19.0% of those < 50 years of age, with serious TEAEs in 34.4% and 19.1% of participants, respectively. Discontinuation due to TEAEs occurred in 4.2% and 1.1% of participants in the ≥ 50 and < 50 years of age groups, respectively, with 6 deaths (6.3%) among those ≥ 50 years of age and 2 deaths (0.4%) among those < 50 years of age. In the virologically suppressed cohort, TEAEs were observed in 78.7% of participants ≥ 50 years of age and in 77.2% of those < 50 years of age, with study drug–related TEAEs in 12.9% and 12.5% of each group, respectively. Grade 3 or 4 TEAEs were seen in 7.3% and 4.7% of participants ≥ 50 and < 50 years of age, respectively, with serious TEAEs in 8.7% and 4.7%. Discontinuation due to TEAEs occurred in 1.8% of participants ≥ 50 years of age and 0.9% of those < 50 years of age, with 2 deaths reported in each age group (0.4% and 0.3%, respectively; Table 4).

**Table 4:** TEAEs

TEAE, *n* (%)	Treatment-naïve cohort		Virologically suppressed cohort	
	≥ 50 years of age	< 50 years of age	≥ 50 years of age	< 50 years of age
	*n* = 96	*n* = 538	*n* = 450	*n* = 640
Any TEAE ^a^	90 (93.8)	514 (95.5)	354 (78.7)	494 (77.2)
Study drug–related TEAEs	25 (26.0)	153 (28.4)	58 (12.9)	80 (12.5)
Any grade 3 or 4 TEAEs	30 (31.3)	102 (19.0)	33 (7.3)	30 (4.7)
Study drug–related grade 3 or 4 TEAEs	4 (4.2)^b^	5 (0.9)^c^	3 (0.7)	4 (0.6)
Any serious TEAE	33 (34.4)	103 (19.1)	39 (8.7)	30 (4.7)
Study drug–related serious TEAEs	2 (2.1)^d^	3 (0.6)^e^	1 (0.2)	1 (0.2)
Study drug discontinuation due to TEAEs	4 (4.2)^f^	6 (1.1)^g^	8 (1.8)	6 (0.9)
Death	6 (6.3)^h^	2 (0.4)^i^	2 (0.4)	2 (0.3)

*COVID* Coronavirus, *TEAE* Treatment-emergent adverse event

^a^Safety through the end of the study. ^b^Due to atrial flutter, dizziness, and acute pancreatitis (in the same participant); and abdominal pain, atypical chest pain, and elevated liver enzyme levels (*n* = 1 each). ^c^Due to abdominal distention, diarrhea, generalized tonic-clonic seizure, osteoporosis, and suicide attempt (*n* = 1 each). ^d^Due to atrial flutter, acute pancreatitis, and dizziness (in the same participant); and chest pain (*n* = 1). ^e^Due to generalized tonic-clonic seizure, spontaneous abortion, and suicide attempt (*n* = 1 each). ^f^Due to cardiac arrest, chest pain, COVID, and obesity (*n* = 1 each). ^g^Due to abdominal distension, dyspepsia, toxicity due to various agents, intervertebral discitis, and tension headache (*n* = 1 each). ^h^Due to cardiac arrest (*n* = 2) and hypertensive heart disease with congestive heart failure, poorly differentiated gastric adenocarcinoma, COVID, and drug toxicity (*n* = 1 each). ^i^Due to hemorrhagic hypovolemia (self-inflicted) and an unknown cause (*n* = 1 each). For the treatment-naïve cohort, data on TEAEs were collected through Week 240 (end of study). For the virologically suppressed cohort, data on TEAEs were collected through Week 48

### Treatment-emergent diabetes and hypertension

Treatment-emergent (events that occur while on study) diabetes and hypertension through Week 240 for the treatment-naïve cohort and through Week 48 for the virologically suppressed cohort are shown in Table 5. From baseline to Week 240, treatment-emergent diabetes was observed in 5.1% of participants ≥ 50 years of age and 1.7% of those < 50 years of age (*P* = 0.08). Treatment-emergent hypertension was reported in 19.6% of participants ≥ 50 years of age and 12.5% of those < 50 years of age (*P* = 0.19). At Week 48 in the virologically suppressed cohort, the incidence of treatment-emergent diabetes was 1.1% in participants ≥ 50 years of age and 1.3% in those < 50 years of age (*P* = 1.00), while treatment-emergent hypertension was observed in 5.2% of participants ≥ 50 years of age and 2.6% of those < 50 years of age (*P* = 0.07). No significant difference in rates of treatment-emergent diabetes or hypertension was found between age groups in either cohort (Table 5).

**Table 5:** Treatment-emergent diabetes and hypertension

	Treatment-naïve cohort				*P* value	Virologically suppressed cohort				*P* value^b^
	≥ 50 years of age		< 50 years of age			≥ 50 years of age		< 50 years of age		
	*n* = 538		*n* = 450			*n* = 96		*n* = 538		
	*n* (%)	Participants with available data, *n*	*n* (%)	Participants with available data, *n*		*n* (%)	Participants with available data, *n*	*n* (%)	Participants with available data, *n*	
Treatment-emergent diabetes^a^	4 (5.1)	78	9 (1.7)	515	0.08	4 (1.1)	374	8 (1.3)	602	1.00
Treatment-emergent hypertension ^a^	10 (19.6)	51	61 (12.5)	489	0.19	14 (5.2)	268	14 (2.6)	529	0.07

For the treatment-naïve cohort, treatment-emergent diabetes and hypertension data were collected through Week 240 (end of study). For the virologically suppressed cohort, treatment-emergent diabetes and hypertension data were collected through Week 48

^a^Participants with a medical history of diabetes and hypertension were excluded. ^b^Calculated by Fisher exact test

## Discussion

This analysis demonstrated that B/F/TAF offers durable efficacy and a favorable safety profile for PWH ≥ 50 years of age, making it as a suitable option for long-term treatment in this growing population. High rates of virologic suppression were maintained in both the treatment-naïve and virologically suppressed cohorts, with no significant difference between older and younger participants. These findings highlight B/F/TAF’s ability to provide sustained virologic control while addressing the unique treatment needs of older adults. Notably, adherence rates remained high among older participants [22, 23], reinforcing the regimen’s practicality as a once-daily, single-tablet regimen that is well-suited to complex patient needs. This analysis includes older PWH (≥ 50 years of age), with a median age of 55 years in the treatment-naïve cohort and 56 years in the virologically suppressed cohort and provides valuable insights into baseline characteristics and treatment outcomes. The inclusion of older PWH helps address a critical gap in understanding the efficacy and safety of B/F/TAF among this population.

B/F/TAF has previously demonstrated durable, long-term viral suppression in diverse populations, including in those with significant comorbidities [19]. Its high barrier to resistance and flexibility in adherence make it particularly well-suited for older adults, especially those who may face additional adherence challenges due to polypharmacy or cognitive changes [24, 25]. In the treatment-naïve cohort of this study, B/F/TAF achieved high rates of viral suppression, even among participants with lower adherence over a prolonged follow-up period, highlighting the regimen’s durability in observational settings where suboptimal adherence often occurs. The low incidence of drug-related adverse events further supports the regimen’s suitability for older PWH, who may have an increased risk of adverse events due to comorbidities and concomitant medications.

Immunologic outcomes were similarly robust across age groups, with older participants demonstrating CD4 T-cell gains that were comparable with those of younger participants. This is especially relevant for older PWH, who are at a greater risk for infections and other immune-related complications due to age-associated immune decline.

Safety outcomes were favorable overall, with low study drug discontinuation due to TEAEs and study drug related grade 3 or 4 TEAEs. Although serious adverse events were more frequent among older participants, most were not drug-related, reflecting the greater comorbidity burden in this population. Importantly, B/F/TAF had minimal impact on weight, lipid profiles, and other metabolic markers, including diabetes mellitus and hypertension rates, in both the treatment-naïve and virologically suppressed cohorts, which is particularly beneficial for older adults who are at an elevated baseline risk for cardiovascular conditions.

Dyslipidemia is a recognized contributor to cardiovascular risk in PWH > 50 years of age, and certain ART regimens have been linked to adverse effects on lipid profiles [26, 27]. In contrast, B/F/TAF is considered metabolically neutral and is recommended as a first-line treatment for HIV-1, with a favorable safety profile for individuals at an elevated cardiovascular risk. In this study, the higher proportion of participants ≥ 50 years of age who initiated lipid-modifying agents likely reflects their increased baseline cardiovascular risk. Our analysis suggests that participants with adverse baseline lipid levels may benefit most from switching to B/F/TAF from prior regimens that include abacavir or boosted protease inhibitors, underscoring its suitability for older PWH who have dyslipidemia and cardiovascular concerns [28, 29]. Notably, recent guideline updates informed by the REPRIEVE trial emphasize the importance of comprehensive cardiovascular risk management in PWH, which includes lipid-modifying therapy as an essential component [30, 31].

Renal and bone safety are also critical considerations in the long-term management of PWH, particularly in older adults at risk for age-related declines in renal function and bone health [12–15, 21]. Chronic kidney disease is more common in PWH ≥ 50 years of age; yet, in this analysis, B/F/TAF demonstrated minimal nephrotoxic impact, with changes in eGFR that were comparable across age groups. These changes showed minimal declines, with no significant differences observed between participants ≥ 50 and < 50 years of age in both the treatment-naïve and virologically suppressed cohorts. This is consistent with the known inhibition of organic cation transporter–2 and tubular creatinine secretion by bictegravir, as previously reported. B/F/TAF also exhibited a neutral effect on BMD, an important finding given that HIV infection and some ART regimens have been associated with an increased risk of osteoporosis and fractures, especially in older adults [14, 32]. BMD changes were minimal in both the hip and spine across both age groups.

In addition to renal and bone safety, metabolic outcomes were also evaluated, such as the emergence of type 2 diabetes mellitus and hypertension during the study. The incidence rate of both conditions was low and comparable across age groups, with a modestly higher occurrence in older, treatment-naïve participants but no significant difference in the virologically suppressed cohort. These findings underscore B/F/TAF’s favorable metabolic profile, as it does not appear to exacerbate the risk of dysglycemia or hypertension, especially in virologically suppressed PWH.

This study has limitations, including the underrepresentation of participants > 65 years of age (particularly in the treatment-naïve group, where only 5 individuals were included) and the lack of data for those > 80 years of age, which limits the ability to fully assess the safety profile in older age groups. Additionally, the open-label design of Studies 1878 and 1961, as well as the underrepresentation of participants with advanced immunosuppression and women, may affect the generalizability of the findings. Greater efforts to enroll women and other underserved populations in large phase 3 trials are needed to ensure that study results are broadly applicable. Nevertheless, the results align well with prior randomized trials of B/F/TAF, supporting the applicability of these findings to a broader population within the studied age range.

In conclusion, B/F/TAF demonstrated sustained efficacy, a favorable safety profile, and high tolerability in PWH ≥ 50 years of age, including in both the treatment-naïve and virologically suppressed cohorts, underscoring its value as an optimal treatment option for managing HIV in aging populations. These findings contribute to the growing evidence that supports B/F/TAF as an effective ART that addresses both HIV-related needs and the additional health complexities faced by older adults.

## Supplementary Information

Supplementary Material 1.

## Data Availability

Gilead Sciences shares anonymized individual participant data upon request or as required by law or regulation with qualified external researchers based on submitted curriculum vitae that reflect no conflicts of interest. The request proposal must also include a statistician. Approval of such requests is at Gilead Science’s discretion and is dependent on the nature of the request, the merit of the research proposed, the availability of the data, and the intended use of the data. Data requests should be sent to datasharing@gilead.com.

## Abbreviations

ART: Antiretroviral therapy
B/F/TAF: Bictegravir/emtricitabine/tenofovir alafenamide
BMD: Bone mineral density
CI: Confidence interval
eGFR: Estimated glomerular filtration rate
FDA: US Food and Administration
HDL: High-density lipoprotein
HIV: Human immunodeficiency virus
LDL: Low-density lipoprotein
LSMD: Least squares mean difference
M= E: Missing= excluded
M= F: Missing= failure
PWH: People with HIV
Q: Quartile
SD: Standard deviation
SMQ: Standardized*Medical Dictionary for Regulatory Activities* query
TAF: Tenofovir alafenamide
TC: Total cholesterol
TDF: Tenofovir disoproxil fumarate
TEAE: Treatment-emergent adverse event
