# Supplementary material for: Efficacy and safety of B/F/TAF in treatment-naïve and virologically suppressed people with HIV ≥ 50 years of age: integrated analysis from six phase 3 clinical trials
Source: BMC Infect Dis. 2025 Aug 22;25:1061. doi: 10.1186/s12879-025-11476-3 (PMC12374459; doi:10.1186/s12879-025-11476-3)
Supplement: Supplementary file 1 — Supplementary Material 1. [file 12879_2025_11476_MOESM1_ESM.docx]

**SUPPLEMENTAL MATERIAL**

**Supplemental Figure 1. Study design for the treatment-naïve (A) and virologically suppressed (B) cohorts.**


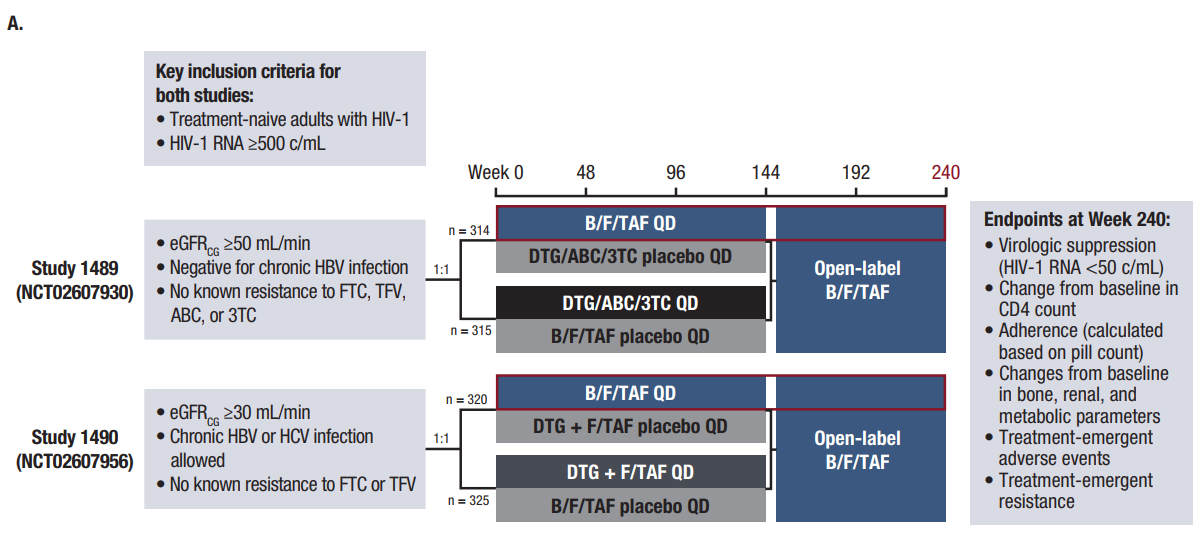


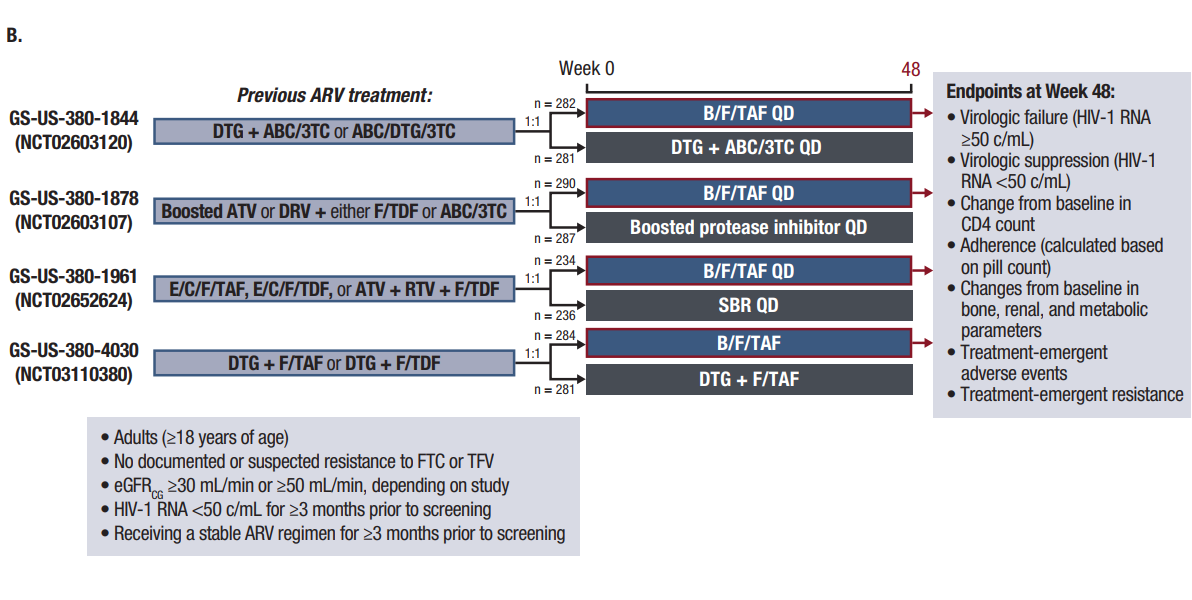


3TC, lamivudine; ABC, abacavir; ARV, antiretroviral; ATV, atazanavir; B, bictegravir; c, copies; C, cobicistat; DRV, darunavir; DTG, dolutegravir; E, elvitegravir; eGFR_CG_, estimated glomerular filtration rate by Cockcroft-Gault; F, emtricitabine; FTC, emtricitabine; HBV, hepatitis B virus; HCV, hepatitis C virus; HIV, human immunodeficiency virus–1; QD, once daily; RTV, ritonavir; SBR, stable baseline regimen; TAF, tenofovir alafenamide; TDF, tenofovir disoproxil fumarate; TFV, tenofovir.

**Supplemental Table 1. Distribution of BMI at Week 240 (TN Cohort) and Week 48 (VS Cohort)**

| **Age Group** | | **TN Cohort** | | | | | | **VS Cohort** | | | | | |  |
| --- | --- | --- | --- | --- | --- | --- | --- | --- | --- | --- | --- | --- | --- | --- |
|  |  | **≥ 50 Years Old** | | | **< 50 Years Old** | | | **≥ 50 Years Old** | | | **< 50 Years Old** | | |  |
| **Baseline BMI Category** | | <25 kg/m^2^ | 25 - <30 kg/m^2^ | ≥30 kg/m^2^ | <25 kg/m^2^ | 25 - <30 kg/m^2^ | ≥30 kg/m^2^ | <25 kg/m^2^ | 25 - <30 kg/m^2^ | ≥30 kg/m^2^ | <25 kg/m^2^ | 25 - <30 kg/m^2^ | ≥30 kg/m^2^ |  |
| **N** | | 30 | 38 | 28 | 277 | 170 | 91 | 154 | 178 | 118 | 292 | 210 | 138 |  |
| **At Week 240: <25 kg/m^2^** | | 10 (50.0%) | 2 (7.4%) | 0 | 104 (58.1%) | 7 (6.0%) | 0 | 105 (72.9%) | 14 (8.1%) | 0 | 219 (79.9%) | 9 (4.5%) | 0 |  |
| **At Week 240: 25-<30 kg/m^2^** | | 10 (50.0%) | 20 (74.1%) | 2 (9.5%) | 66 (36.9%) | 66 (56.9%) | 7 (10.3%) | 39 (27.1%) | 140 (80.9%) | 10 (8.9%) | 55 (20.1%) | 168 (83.2%) | 8 (6.0%) |  |
| **At Week 240: ≥30 kg/m^2^** | | 0 | 5 (18.5%) | 19 (90.5%) | 9 (5.0%) | 43 (37.1%) | 61 (89.7%) | 0 | 19 (11.0%) | 102 (91.1%) | 0 | 25 (12.4%) | 125 (94.0%) |  |
| **Missing** | | 10 | 11 | 7 | 98 | 54 | 23 | 10 | 5 | 6 | 18 | 8 | 5 |  |
| BMI = body mass index; n = number of participants in each baseline BMI category. | | | | | | | | | | | | | | |
| Percentages are calculated using the number of participants with no missing values in a given baseline BMI category within an age group.  “Missing” indicates participants without available data for BMI at follow-up. | | | | | | | | | | | | | |  |
| P-values for comparison of BMI shifts by age group: | | | | | | | | | | | | | | |
| Week 240 (Naïve cohort): p = 0.3377 | | | | | | | | | | | | | |  |
| Week 48 (Switch cohort): p = 0.9253 | | | | | | | | | | | | | |  |
